# Heart Failure With Cardiogenic Shock as a Manifestation of Untreated POEMS (Polyneuropathy, Organomegaly, Endocrinopathy, Monoclonal Protein, Skin Changes) Syndrome

**DOI:** 10.7759/cureus.18046

**Published:** 2021-09-17

**Authors:** Mandi Abdelahad, Ryan Pearson, Bryant Mauri, Kira Fenton, Cristina Savu

**Affiliations:** 1 Internal Medicine, Nova Southeastern University Dr. Kiran C. Patel College of Osteopathic Medicine, Fort Lauderdale, USA; 2 Internal Medicine, Broward Health Medical Center, Fort Lauderdale, USA

**Keywords:** poems syndrome, castleman disease, heart failure, pulmonary hypertension, hematology

## Abstract

POEMS syndrome (polyneuropathy, organomegaly, endocrinopathy, monoclonal protein, skin changes) is a rare paraneoplastic syndrome due to a plasma cell disorder. Diagnosis requires peripheral neuropathy and a monoclonal plasma cell disorder along with one major and one minor criteria, but cardiac manifestations are uncommon. The pathogenesis of POEMS syndrome is not well understood but it is thought to involve overproduction of proinflammatory cytokines, such as vascular endothelial growth factor (VEGF), interleukin-1 beta (IL-1B), interleukin-6 (IL-6), and tumor necrosis factor-alpha (TNF-alpha). POEMS syndrome commonly presents in the fifth to sixth decade of life, mainly in non-Hispanic Caucasian individuals, and affects more men than women (2:1). We report a unique case of a 28-year-old African American female with a history of POEMS syndrome and a new diagnosis of dilated, non-ischemic cardiomyopathy and New York Heart Association (NYHA) class IV, stage D heart failure with an ejection fraction (EF) of 30% as a result of the natural progression of her untreated POEMS syndrome.

## Introduction

POEMS syndrome (polyneuropathy, organomegaly, endocrinopathy, monoclonal protein, skin changes) is a rare paraneoplastic syndrome due to an underlying plasma cell disorder. Mandatory criteria required for diagnosis include peripheral neuropathy and monoclonal plasma cell disorder, almost always of the lambda light chain type [1]. In addition to the acronym and the two mandatory criteria, patients can have one or more of the following features: osteosclerotic lesions, Castleman disease (angiofollicular lymph node hyperplasia), increased levels of serum vascular endothelial growth factor (VEGF), extravascular volume overload, thrombocytosis, erythrocytosis, and papilledema [1]. The pathogenesis is not well understood but is thought to involve chronic overproduction of proinflammatory cytokines, such as VEGF, interleukin-1 beta (IL-1B), interleukin-6 (IL-6), and tumor necrosis factor-alpha (TNF-alpha), possibly explaining the variety of symptom manifestations in such patients [2].

POEMS syndrome commonly presents in the fifth to sixth decade and affects males at a higher proportion than females, with overall median survival of 13.7 years after diagnosis [1]. The syndrome was initially thought to be most common in Japanese populations but is now being reported in China, France, and the United States. The reason for this new broadened incidence is thought to be in the setting of increased awareness of the syndrome [1]. We present the case of a 28-year-old African American female with a history of POEMS syndrome in addition to an unfortunate newly diagnosed dilated, non-ischemic cardiomyopathy and New York Heart Association (NYHA) class IV, stage D heart failure with an ejection fraction (EF) of 30%. This case is unique given the patient is a young African American female with POEMS syndrome in the setting of acute heart failure.

## Case presentation

A 28-year-old African American female with a past medical history of POEMS syndrome presented with recurring episodes of nausea, vomiting, and non-bloody, non-bilious (NBNB) diarrhea for months that are not associated with oral intake. The patient denied any recent travel or sick contacts. She also denied any fevers, chills, chest pain, shortness of breath, or abdominal pain.

**Figure 1:**
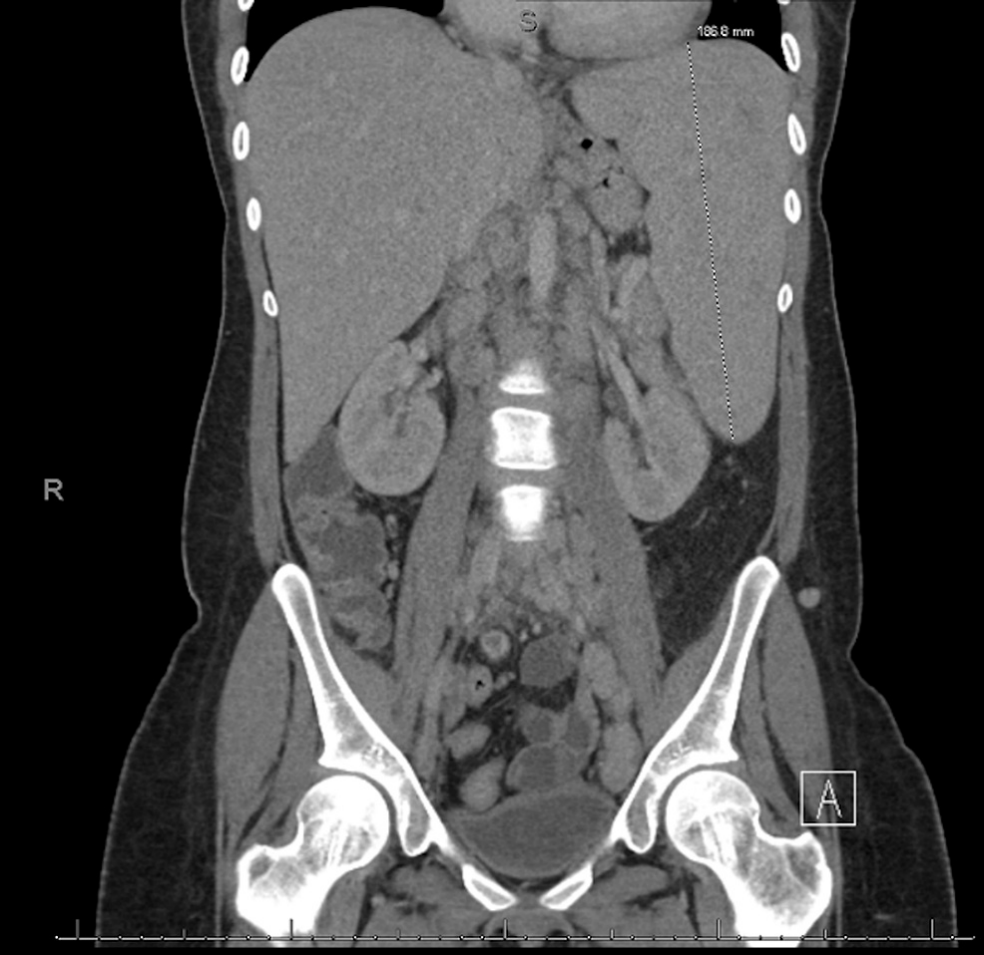
CT abdomen and pelvis with IV contrast showing splenomegaly (186.8 mm)

The patient was initially diagnosed with Castleman disease two years ago via an axillary lymph node biopsy and was offered chemotherapy but declined for concern of side effects and financial burden. A bone marrow biopsy later revealed a hypocellular marrow at 40%. After a careful review amongst a multidisciplinary specialty tumor board, the patient was subsequently diagnosed with POEMS syndrome. At the time of diagnosis, the patient met diagnostic criteria which included: progressive neuropathy, elevated VEGF at 304 pg/mL (reference range: 31-86 pg/mL), splenomegaly at 186.8 millimeters (mm) (reference range: 76-131 mm), left para-aortic lymph node measuring 2.3 centimeters (cm) (reference range: 1.1 cm), left iliac chain lymph node measuring 2.7 cm (reference range: 0.7 cm), 2+ pitting edema bilaterally and bilateral foot skin ulcerations (Figure 1). Urine protein electrophoresis (UPEP) results indicated a monoclonal spike (M-spike) but was negligible to quantify (urine protein electrophoresis/urine immunofixation electrophoresis {UPEP/UIFE}: k/L 526/147 = 3.58). Serum protein electrophoresis (SPEP) results did not indicate an M-spike (serum protein electrophoresis/serum immunofixation electrophoresis {SPEP/SIFE}: k/L 78.2/71.2 = 1.09). Hematology recommended starting dexamethasone and lenalidomide (Revlimid) while waiting for stem cell transplant. Unfortunately, the patient again refused further treatment.

**Figure 2:**
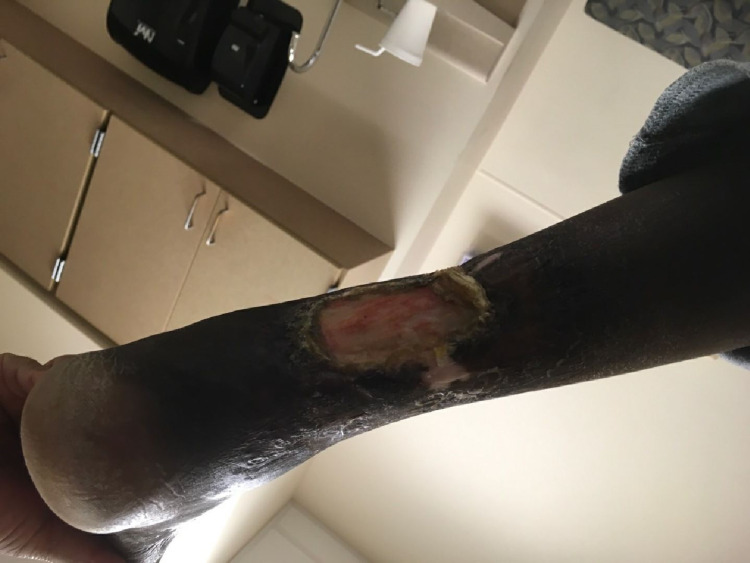
Chronic stage III ulcer on the posterior aspect of the left lower extremity

**Figure 3:**
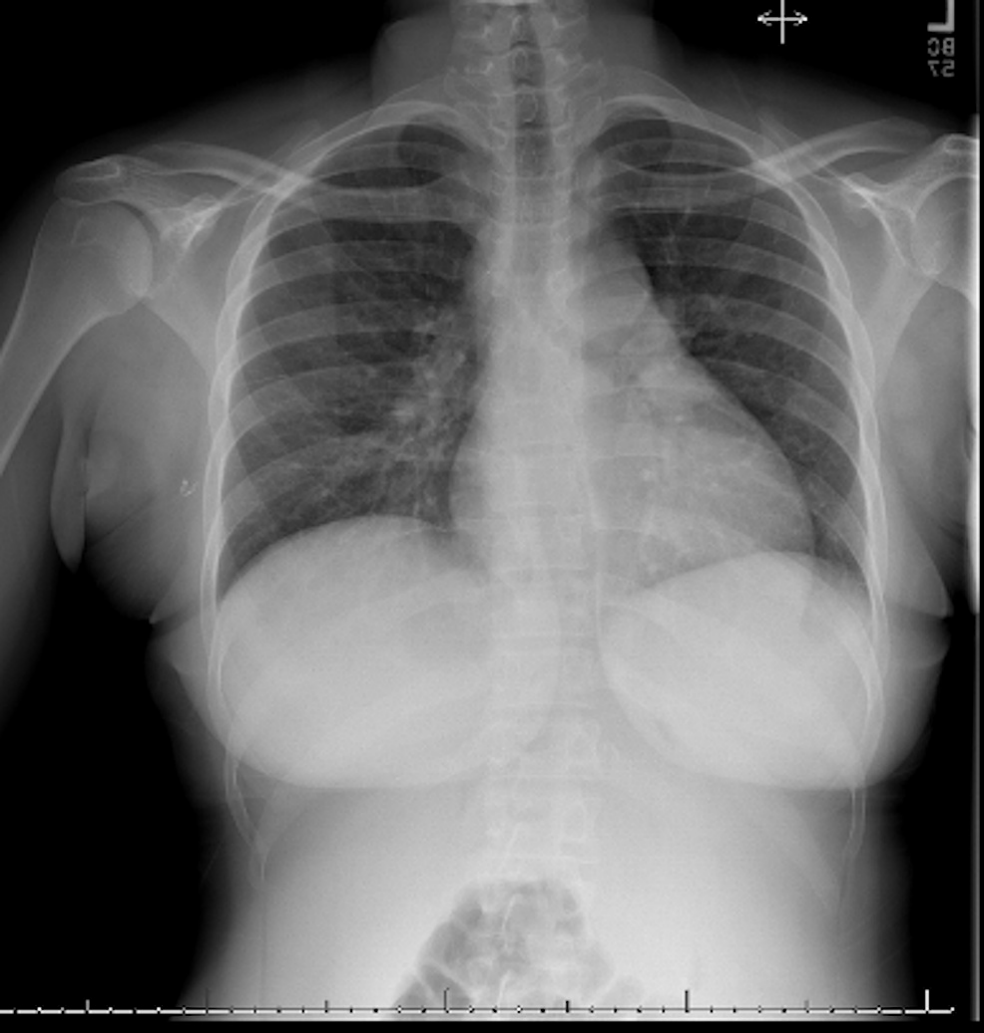
Cardiomegaly on chest x-ray

On admission, the patient was febrile at 101.5°F (38.6°C), blood pressure was 95/44 mmHg, and tachycardic at 133 beats per minute (bpm). Physical examination revealed an unremarkable cardiopulmonary examination, including the absence of jugular venous distention (JVD) or lower extremity edema. She had non-tender hepatosplenomegaly, non-tender bilateral lymphadenopathy to the submandibular, axillary, and inguinal regions. She had chronic stage III ulcer on the posterior aspect of the left lower extremity (Figure 2). Complete blood count (CBC) revealed white blood cell (WBC) count of 9.63 × 10^9^ WBCs per microliter (WBCs/mcL) (reference range: 4.5-11.0 × 10^9^ WBCs/mcL) microcytic, hypochromic anemia with a hemoglobin of 6.3 grams per deciliter (g/dL) (reference range: 12.0-15.5 g/dL). Ferritin level was >35,000 micrograms per liter (mcg/L) (reference range: 11-307 mcg/L), procalcitonin was >100 nanograms per milliliter (ng/mL) (reference range <0.15 ng/mL). Lactic acid was 1.3 (reference range: 0.5-1 mmol/L) on admission and increased to 5.8 mmol/L within 24 hours. Repeat SPEP was consistent with an increase in polyclonal gamma globulins and a negative M spike. Peripheral blood flow cytometry report was negative for any acute hematological processes. When compared to a previous study from 2020, the admission computed tomography (CT) abdomen and pelvis with intravenous (IV) contrast was significant for splenomegaly with slightly worsened adenopathy (Figure 1). Chest x-ray showed cardiomegaly (Figure 3). The patient was admitted for anemia and septicemia with an unclear etiological source.

**Figure 4:**
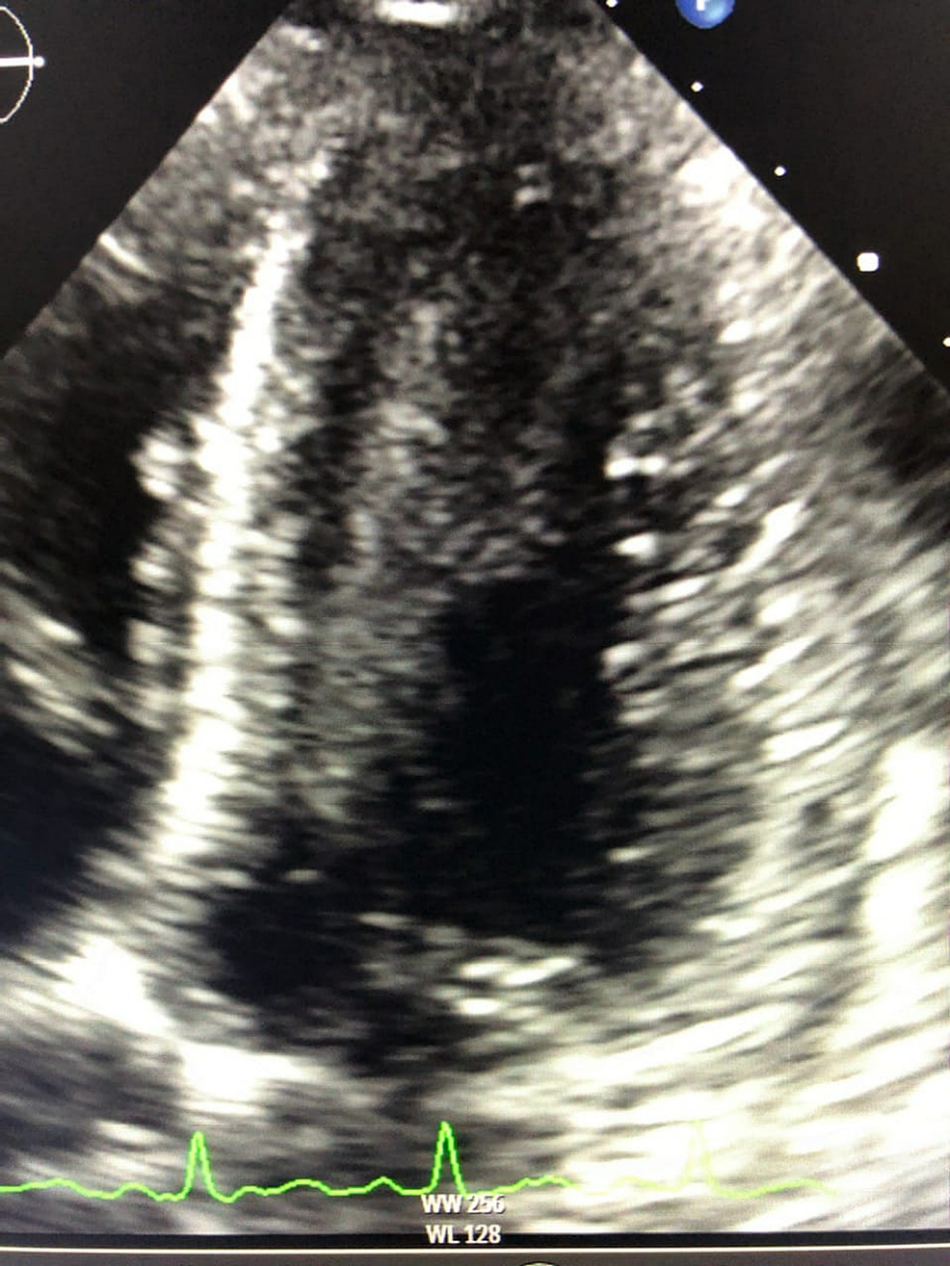
Moderate to severe global hypokinesis of the LV on echocardiogram LV: left ventricle

The patient’s febrile episodes persisted for two days, with a maximum recorded temperature of 103°F (39.4°C). Within 48 hours of admission, her status deteriorated. She developed shock despite fluid resuscitation, requiring higher acuity of care in the intensive care unit (ICU). The patient was placed on norepinephrine, midodrine, IV maintenance fluids, IV methylprednisolone, and antibiotics including vancomycin, Levaquin, Flagyl, and micafungin. CT angiography of the pulmonary arteries showed no pulmonary embolism. Echocardiogram (ECHO) revealed a moderate to severely reduced left ventricular (LV) systolic function with EF of 25-30%, grade II diastolic dysfunction, moderate to severe global hypokinesis of the LV, elevated right ventricular systolic pressure (RVSP) of 52 mmHg and a flattened septum consistent with RV pressure/volume overload, yet, physical examination still shows no signs of JVD (Figure 4).

Right and left cardiac catheterizations revealed normal coronary arteries, a dilated, non-ischemic cardiomyopathy with an EF of 30%, and moderate pulmonary hypertension, likely from pulmonary etiology (Table 1). Based on the hemodynamic pressure readings, results were inconclusive for a constrictive process and the patient was classified as NYHA class IV, stage D heart failure.

**Table 1:** Right heart catheterization measurements

Pulmonary capillary wedge pressure (PCWP)	13/12 mmHg with a mean of 16 mmHg
Pulmonary artery (PA)	54/25 mmHg with a mean of 35 mmHg
Right ventricle (RV)	53/3 with RV end-diastolic pressure (RVEDP) of 12 mmHg
Right atrium (RA)	11/10 mmHg with a mean of 28 mmHg
Cardiac output (CO)	5.2 liters/min (L/min)
Cardiac index (CI)	2.7 L/min/m^2^
Systemic vascular resistance (SVR)	22.6 hybrid reference units (HRU)
Pulmonary vascular resistance (RVR)	3.45 HRU

IV furosemide was used for diuresis and the shock and symptoms improved. Vasopressor support was tapered and febrile episodes ceased. All cultures obtained on the admission were negative, and thus antibiotics were discontinued. Once the patient was transferred out of the ICU and hemodynamically stable on the medical floor, cardiology started a heart failure regimen of furosemide, valsartan, and metoprolol succinate that was continued in the outpatient setting. Spironolactone was not initiated as it was not needed at the time due to improvement of clinical status. Hematology recommended dexamethasone and Revlimid. After an extensive discussion emphasizing the risks and benefits of therapy, the patient agreed to begin chemotherapy in the outpatient setting and was willing to reconsider stem cell transplant once her EF improved.

## Discussion

POEMS syndrome is a rare, multisystemic disease where the acronym refers to polyneuropathy, organomegaly, endocrinopathy, monoclonal protein, and skin changes [1]. The syndrome presents with a wide spectrum of clinical manifestations, making it difficult to properly diagnose. Diagnosis requires fulfilling two mandatory criteria, one or more major criteria and one or more minor criteria (Table 2) [1]. In a Mayo Clinic study of 99 patients with POEMS syndrome, the median age was 51 years, 89% were non-Hispanic Caucasian patients and 63% were males [3]. This report presents a rare case of a 28-year-old African American female with POEMS syndrome and newly diagnosed dilated, non-ischemic cardiomyopathy with an EF of 30%. The pathogenesis is thought to involve an overproduction of proinflammatory cytokines, such as VEGF, IL-1B, IL-6, and TNF-alpha. Overactivation of these inflammatory cytokines acting like endogenous pyrogens could potentially explain our patient’s episodes of recurrent fevers.

**Table 2:** Criteria for the diagnosis of POEMS syndrome

Mandatory major criteria	Polyneuropathy (typically demyelinating)
	Monoclonal plasma cell-proliferative disorder (almost always)
Other major criteria (one required)	Castleman disease
	Sclerotic bone lesions
	Vascular endothelial growth factor elevation
Minor criteria	Organomegaly (splenomegaly, hepatomegaly, or lymphadenopathy)
	Extravascular volume overload (edema, pleural effusion, or ascites)
	Endocrinopathy (adrenal, thyroid, pituitary, gonadal, parathyroid, pancreas)
	Skin changes (hyperpigmentation, hypertrichosis, glomeruloid hemangiomata, plethora, acrocyanosis, flushing, white nails)
	Papilledema
	Thrombocytosis/polycythemia
Other symptoms and signs	Clubbing, weight loss, hyperhidrosis, pulmonary hypertension/restrictive lung disease, thrombotic diatheses, diarrhea, low vitamin B12 values

Our patient fulfilled both mandatory criteria of polyneuropathy and monoclonal protein spike, fulfilled major criteria with Castleman disease and elevated VEGF, and fulfilled minor criteria significant for splenomegaly, cardiomegaly, edema, and skin changes to support the diagnosis of POEMS syndrome. Castleman disease, a rare lymphoproliferative disease, has been reported in up to 30% of patients with POEMS, but this data is inconclusive as many patients do not undergo lymph node biopsy [3]. Castleman is distinguished from POEMS by demonstrating the lack of peripheral neuropathy and a plasma cell dysplasia and in these patients, a diagnosis of Castleman disease variant of POEMS syndrome can be considered if they have other major and minor criteria for POEMS syndrome [4].

Pulmonary hypertension and congestive heart failure may be included as uncommon features of POEMS syndrome on therapy, as seen in our patient [3,5]. In the Mayo Clinic study, only five patients had pulmonary hypertension and three patients had congestive heart failure and cardiomyopathy [3,5]. Literature review revealed a successful case of a 55-year-old female with POEMS syndrome who presented with LV systolic dysfunction that was treated with thalidomide plus dexamethasone with marked improvement of systolic dysfunction [6]. The mechanism underlying the left ventricular systolic dysfunction may involve extracellular edema due to an overexpression of VEGF in the wall of the myocardium, which may be an explanation for our patient’s presentation as well [7]. The etiology of shock in our patient was initially thought to be distributive in nature, but it was concluded that the shock was cardiogenic, likely in the setting of a fluid overloaded state inducing stage D heart failure. The patient had an ECHO performed a year prior which was normal, suggesting the new results showed a significant decline in function from baseline.

There is no standard treatment for POEMS, but major therapeutic approach includes steroids, chemotherapy, radiation therapy for limited disease, and hematopoietic cell transplant (HCT) for disseminated disease. Multiple studies have demonstrated clinical improvement with lenalidomide and dexamethasone in over 70% with 60-75% progression-free at a three-year follow-up [8]. In a study of 59 patients treated with autologous HCT using peripheral blood stem cells, clinical improvement was rapid and nearly universal in these patients [9]. At a median follow-up time of 45 months, the five-year overall and progression-free survival rates were 94% and 75%, respectively, for this population. Fourteen patients continued with progressive disease post-transplant but manifested only with hematologic abnormalities, radiographic findings, or increased VEGF levels and none had clinical symptoms, thus stressing the importance of prompt recognition and treatment of this syndrome [9].

## Conclusions

POEMS syndrome (polyneuropathy, organomegaly, endocrinopathy, monoclonal protein, skin changes) is a rare paraneoplastic syndrome due to an underlying plasma cell disorder. Given our patient’s unique age, sex, and ethnicity, even rarer presentation of untreated, cardiogenic shock, manifesting as a new diagnosis of NYHA class IV, stage D heart failure with an EF of 30%. The purpose of this case is to provide increased awareness of POEMS syndrome and to take into consideration its distinguishing criteria, especially when evaluating patients in the context of multisystemic clinical manifestations and features. Early recognition is warranted as treatment with steroids, chemotherapy, and HCT shows an overall better quality of life for patients to prevent the natural progression of untreated disease.
